# Left Atrial Structural and Functional Changes After Pulmonary Vein Isolation Using Different Energy Sources

**DOI:** 10.3390/s26134103

**Published:** 2026-06-28

**Authors:** Jonasz Kozielski, Monika Olczyk, Ewa Świerżyńska-Wodarska, Michał Orczykowski, Bronisław Sulkowski, Łukasz Szumowski, Maciej Sterliński, Marek Szołkiewicz

**Affiliations:** 1 Department of Cardiology and Interventional Angiology, Kashubian Center for Heart and Vascular Diseases, Pomeranian Hospitals, 84-200 Wejherowo, Poland; monika_rybarczyk@interia.pl (M.O.); e.mars@wp.pl (M.S.); 2 1st Department of Arrhythmia, The Cardinal Stefan Wyszynski, National Institute of Cardiology, 04-628 Warsaw, Poland; eswierzynska@ikard.pl (E.Ś.-W.); morczykowski@ikard.pl (M.O.);; 3 Department of Mathematics, Statistics & Informatics, Faculty of Pharmacy, Medical University of Gdansk, 80-210 Gdansk, Poland

**Keywords:** atrial fibrillation, catheter ablation, echocardiography

## Abstract

***Background:*** Pulmonary vein isolation (PVI) is an established treatment for atrial fibrillation (AF); however, its impact on long-term left atrial (LA) structural and mechanical remodeling may differ between ablation techniques. ***Objectives:*** The aim of this study is to assess changes in LA structure and function after PVI using cryoballoon ablation (CBA) and radiofrequency catheter ablation (RFCA). ***Methods:*** Forty-two patients undergoing PVI were prospectively analyzed (CBA, *n* = 28; RFCA, *n* = 14). Transthoracic echocardiography with speckle-tracking analysis was performed before PVI and at 12-month follow-up. LA size, volumetric indices (LAVI), strain components, and functional parameters were assessed. ***Results:*** Patients undergoing CBA demonstrated greater increases in selected LA structural parameters during follow-up compared with RFCA. Repeated-measures ANOVA revealed a significant group-by-time interaction for LA diameter (*p* = 0.0017), while similar trends were observed for LAA (*p* = 0.0645) and LAS-R (*p* = 0.0547). No significant interaction effects were observed for LAVI-derived parameters or other indices of LA mechanical function. ***Conclusions:*** In this preliminary exploratory study, CBA showed a tendency toward greater structural LA remodeling compared with RFCA during a 12-month follow-up. However, these findings should be interpreted with caution given the limited sample size and population heterogeneity. Larger prospective studies are warranted to validate these observations.

## 1. Introduction

Atrial fibrillation (AF) is the most frequently encountered sustained arrhythmia and remains a major contributor to adverse clinical outcomes, including increased morbidity and mortality [1]. AF is increasingly recognized as a manifestation of atrial cardiomyopathy, in which left atrial (LA) structural remodeling and fibrosis contribute to progressive deterioration of atrial function [2]. It is now well established that the extent of LA interstitial fibrosis broadly reflects the biological and clinical progression of AF, ranging from early structural remodeling in patients with sinus rhythm, through paroxysmal and persistent forms, to long-standing persistent AF. Moreover, interstitial fibrosis typically represents a progressive, multi-step process in anatomical and architectural terms. In most cases, the pulmonary vein outlet region appears to be the predominant initial site of fibrotic involvement [3].

In patients with AF, catheter-based pulmonary vein isolation (PVI) has become a cornerstone of rhythm-control therapy [4] and can partially reverse atrial structural remodeling [5]. Although effective in suppressing arrhythmia, PVI inevitably results in localized atrial myocardial injury [6]. Ablation-related tissue damage may promote myocyte loss and fibrotic replacement, potentially aggravating impairment of LA mechanical performance, particularly in individuals with pre-existing atrial structural remodeling [7]. PVI can be performed using radiofrequency ablation (RFCA), which relies on thermal energy, or cryoballoon ablation (CBA), which achieves tissue injury through cryogenic cooling; both approaches demonstrate comparable long-term efficacy. RFCA and CBA result in tissue injury via different biophysical mechanisms [8]. However, available evidence regarding the extent and nature of ablation-induced myocardial injury by these techniques remains inconsistent. While some studies have suggested more extensive myocardial damage following CBA [9,10,11,12], others have reported comparable [13] or even opposing findings [14] when compared with RFCA.

We sought to examine the influence of RFCA and CBA on LA mechanical function in patients with paroxysmal and persistent AF, focusing on differences between the two modalities.

## 2. Materials and Methods

### 2.1. Study Population

This study was designed as a single-center, prospective observational cohort study conducted at Kashubian Center for Heart and Vascular Diseases in Wejherowo, Poland, between August 2023 and July 2024. The study was approved by the institutional ethical review board nr KB 34/23, and all participants provided written informed consent. We screened 51 consecutive patients who were considered suitable candidates for both CBA and RFCA, accepted the study protocol and provided consent for further contact and follow-up. Patients were selected according to the inclusion and exclusion criteria.

### 2.2. Inclusion and Exclusion Criteria

Patients with paroxysmal, persistent, or long-standing persistent AF were eligible for inclusion if they had maintained sinus rhythm for at least 6 weeks prior to the baseline assessment. All participants were required to be ≥18 years of age and to provide written informed consent. Patients not fulfilling the inclusion criteria were excluded. Additional exclusion criteria comprised pregnancy, the presence of intracardiac thrombus precluding ablation, and documented AF or other atrial arrhythmias either at follow-up or within the 6 weeks preceding evaluation. This time interval was selected to minimize the impact of atrial stunning following atrial tachyarrhythmias, predominantly AF, as previously reported, and to reduce potential bias in follow-up echocardiographic measurements [15,16]. The choice of ablation modality was not randomized and was made according to routine clinical practice at our institution. No predefined clinical, echocardiographic, or anatomical criteria were used to assign patients to CBA or RFCA.

### 2.3. Echocardiography

Transthoracic echocardiography (TTE) was performed using the Vivid E95 system (GE, Milwaukee, WI, USA) to assess LA remodeling by evaluating LA size and functional parameters before and after CA. Echocardiographic parameters were assessed in accordance with the recommendations of the European Association of Cardiovascular Imaging (EACVI) by an experienced echocardiographer and were independently verified by a second physician. LA functional assessment included volumetric and Doppler-derived indices calculated using established formulas, including LA ejection fraction (LAEF), LA active emptying fraction (LA-AE), LA passive emptying fraction (LA-PE), LA expansion index (LA-EI), LA kinetic energy (LAKE), LA function index (LAFI), and the LA stiffness index (LAS-si).

Transesophageal echocardiography (TEE): In all patients, regardless of thromboembolic risk assessed by the CHA_2_DS_2_-VASc score or the type of arrhythmia, TEE was performed within 24 h prior to the procedure to exclude intracardiac thrombus, particularly in the LA appendage. The examination was carried out using the same machine as in TTE. The procedure itself was conducted under mild conscious sedation, achieved with the administration of at least midazolam and/or fentanyl.

### 2.4. Anticoagulation Management

The management of oral anticoagulation during the perioperative period was conducted in the same manner and consisted of withholding the morning dose of anticoagulant medications. The procedure was performed according to standard practice, with administration of unfractionated heparin in doses adjusted to achieve an activated clotting time (ACT) of 300–350 s. The initial loading dose was 120 IU/kg, with half of the dose administered before transseptal puncture. After two TTE procedures—one performed immediately after completion of the procedure and the second 4 h later—patients were restarted on oral anticoagulant therapy according to standard dosing regimens.

### 2.5. Ablation Procedure

Both CBA and RFCA procedures were performed under mild conscious sedation using midazolam and fentanyl. Ultrasound guidance was routinely used to obtain vascular access. Access was achieved exclusively via the right femoral vein. For CBA, three venous punctures were performed to accommodate the coronary sinus catheter, His bundle catheter, and the FlexCath sheath, whereas for RFCA three punctures were also used for placement of the coronary sinus catheter, His bundle catheter, and the Agilis steerable sheath.

CBA was performed using a third-generation ArcticFront (Medtronic). Transseptal puncture was carried out in all patients with the use of the AcQCross transseptal system. All patients were treated with the 28 mm cryoballoon and 20 mm Achieve mapping catheter. The number of freeze applications per PV was operator-dependent, but most patients received one (240 s) freeze application with a target time to isolation (TTI) below <70 s. PV potentials were actively sought using the Achieve mapping catheter and were successfully identified in the majority of patients. When the predefined criteria were not met, an additional 180 s bonus freeze was applied, while no other supplementary applications (such as carina or ridge ablation in the left superior PV region) were performed. Phrenic pacing and monitoring were performed during ablation over the right-sided PVs.

RFCA was started with endocardial surface reconstruction performed using a circular mapping catheter (Advisor™ Lasso FL Mapping Catheter; Abbott, USA, Illinois) for fast anatomical mapping. Ablation was performed with radiofrequency energy using standard irrigated-tip ablation catheter (Tactiflex™, Abbott, USA, Illinois). The standard ablation settings included a preselected power (45 W, 20 s) and a flow rate of 13 mL/min. At the posterior LA wall, power delivery was limited (50 W, 10 s). The standard contact force ranged between 10 and 20 g. Patients presenting with AF were cardioverted. During stable sinus rhythm the electroanatomical map was obtained. A bipolar peak-to-peak electrogram amplitude of <0.5 mV was considered indicative of low-voltage, diseased myocardial tissue. In all patients, standard ostial PVI was performed. Ablation end points were lack of local pace capture and bidirectional conduction block over linear lesions.

### 2.6. Follow-Up

Patients were evaluated on the day of the procedure and at 12-month follow-up. At both time points, all participants underwent a standard 12-lead ECG, TTE, and TEE. Patients reporting symptoms suggestive of arrhythmia, including palpitations or chest discomfort, were instructed to undergo prompt 12-lead ECG or 24 h Holter monitoring and to transmit recordings to the study center. Clinical arrhythmia recurrence and procedural failure were defined as any documented episode of AF or atrial flutter on ECG, or any supraventricular arrhythmia lasting > 30 s on 24 h Holter monitoring. Patients presenting with AF or atrial flutter at the follow-up visit, or with documented arrhythmia recurrence within the 6 weeks preceding follow-up, were excluded from further analysis and considered withdrawn from the study population.

### 2.7. Statistical Analysis

Analyses were conducted using IBM SPSS Statistics, version 23. Continuous variables are presented as mean ± standard deviation (SD), whereas categorical variables are presented as counts and percentages. Data distribution was assessed using the Shapiro–Wilk test and demonstrated no significant departure from normality; therefore, parametric statistical methods were applied. Between-group comparisons were performed using independent-samples *t*-tests, while within-group comparisons were assessed using paired *t*-tests. Categorical variables were compared using Fisher’s exact test. To evaluate longitudinal changes and differences in remodeling trajectories between treatment groups, repeated-measures analysis of variance (ANOVA) was performed, with time (baseline vs. follow-up) as the within-subject factor and ablation modality (CBA vs. RFCA) as the between-subject factor. A two-sided *p*-value < 0.05 was considered statistically significant.

## 3. Results

The study included 51 patients with a mean age of 61 years, of whom 59.6% were male**.** Paroxysmal AF was present in 64% of participants. All patients underwent PVI using either CBA or RFCA performed in 62.75% and 37.25% of cases, respectively. Twelve-month follow-up was available for 47 patients, while 4 patients were lost to follow-up. During the observation period, atrial fibrillation recurrence was documented in five patients, either at the scheduled visit or within the preceding 6 weeks; these patients were excluded from further analysis.

A total of 42 patients were included in the final analysis, of whom 14 underwent RFCA and 28 CBA. Non-response was observed in six patients in the RFCA group and in four patients in the CBA group, corresponding to a higher recurrence rate after RFCA; however, this difference did not reach statistical significance (Fisher’s exact test, *p* = 0.06), although a trend toward increased AF recurrence in the RFCA group was noted.

Patients undergoing CBA and RFCA were comparable with respect to age, sex distribution, body mass index, comorbidity burden, and clinical risk profiles, including CHA_2_DS_2_-VASc and HAS-BLED scores, with no statistically significant differences observed between groups. A noticeable trend difference was noted in the type of AF at baseline. Paroxysmal AF was present in 60.7% of patients in the CBA group, whereas all patients in the RFCA group had paroxysmal AF (86%, *p* = 0.16). No significant differences were observed in AF duration, EHRA symptom class, history of electrical cardioversion, or prior use of antiarrhythmic drugs. With regard to anticoagulation therapy, the overall use of direct oral anticoagulants (DOACs) was high and comparable between groups. However, the distribution of specific DOAC agents differed significantly. Apixaban was more frequently prescribed in the CBA group (53.6% vs. 14.3%, *p* < 0.05), whereas rivaroxaban was more commonly used in patients undergoing RFCA (71.3% vs. 32.0%, *p* < 0.05). The use of dabigatran did not differ significantly between groups, and no patients were treated with vitamin K antagonists at baseline. Baseline demographic and clinical characteristics of the study population are summarized in Table 1.

Baseline echocardiographic characteristics before PVI stratified by ablation modality are presented in Table 2.

No statistically significant differences were observed between the CBA and RFCA groups with respect to LA size or volumetric parameters, including LA diameter, LAVI assessed by both MOD and AL methods, as well as LA maximal, minimal, and pre-A volumes. Similarly, LA mechanical function parameters derived from speckle-tracking analysis did not differ significantly between groups. LA reservoir (LAS-R), conduit (LAS-CD), and contractile (LAS-CT) strain components, as well as LA ejection fraction, LA kinetic energy, LA functional index, LA stiffness index, and average E/e′ ratio were comparable at baseline. A numerical trend toward a higher LA expansion index was observed in the RFCA group compared with the CBA group, although this difference did not reach statistical significance. In addition, LA passive emptying fraction tended to be higher in the RFCA group, representing a borderline difference between groups (*p* = 0.06).

### Echocardiographic Changes After PVI: CBA vs. RFCA

Repeated-measures ANOVA demonstrated distinct patterns of left atrial remodeling following cryoballoon ablation (CBA) and radiofrequency catheter ablation (RFCA).

**Figure 1:**
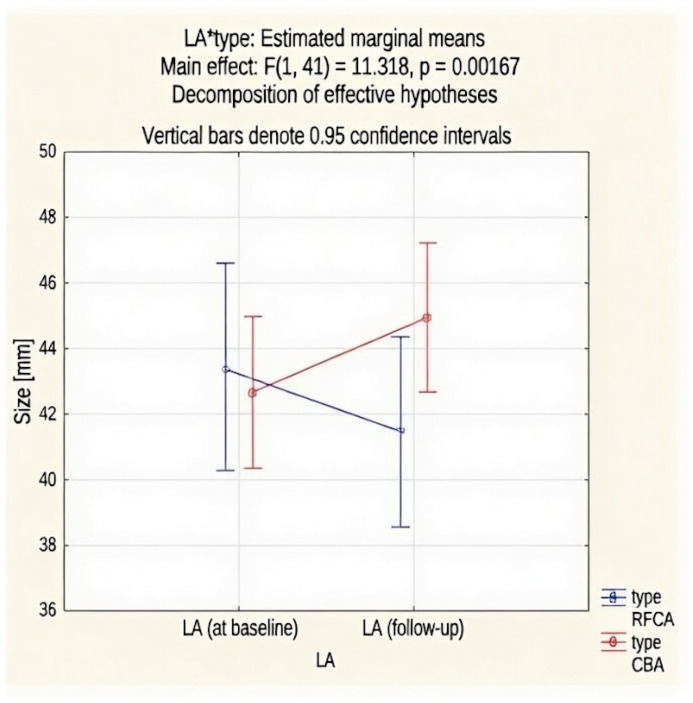
Lef atrium size—ANOVA analyses at baseline and after follow-up after pulmonary vein isolation. Blue—cryoballoon ablation group (CBA), and red—radiofrequency catheter ablation (RFCA) group, assessed before pulmonary vein isolation (PVI) and at 12-month follow-up visit (LA—left atrium).

The most pronounced difference was observed for left atrial diameter, which increased significantly in the CBA group (+2.50 mm, 95% CI: 1.18–3.82) but decreased in the RFCA group (−1.47 mm, 95% CI: −3.79–0.85), resulting in a significant group-by-time interaction (*p* = 0.0017). Similar trends were observed for left atrial area (LAA), which increased after CBA but remained largely unchanged following RFCA (interaction *p* = 0.0645). A trend toward differential functional remodeling was also noted for left atrial reservoir strain (LAS-R), which decreased in both groups, although the decline was more pronounced in RFCA patients (interaction *p* = 0.0547). In contrast, no significant group-by-time interactions were observed for volumetric indices, including LAVI measured by either the modified Simpson or area–length methods. Likewise, no significant differences between treatment modalities were found for LAEF, LAFI, conduit strain, contractile strain, or other derived echocardiographic parameters. Detailed results are presented in Table 3, while the principal remodeling patterns are illustrated in Figure 1.

## 4. Discussion

At 12-month follow-up, patients undergoing CBA demonstrated a tendency toward greater enlargement of selected LA structural parameters, particularly LA diameter, compared with RFCA. However, these changes were not accompanied by deterioration in LA hemodynamic function, and no significant differences between treatment modalities were observed for LAVI-derived indices. LA strain components and emptying indices remained largely unchanged in the overall cohort, whereas RFCA was associated with relative preservation of LA structure and mechanical performance.

Our observations in the RFCA group are largely consistent with previously reported data. Previous studies have demonstrated favorable LA remodeling after RFCA in selected populations; however, these effects appear to be highly dependent on baseline LA size, AF phenotype, and procedural success. The absence of positive remodeling may be partly explained by a relatively high proportion of non-responders (~40%), in whom negative LA remodeling was noted. In contrast, findings in the CBA group are more intriguing, as available evidence suggests functional improvement in responders and stabilization of remodeling in non-responders.

Evidence from other studies indicates that PVI lesions after CBA were characterized by significantly wider and more continuous lesions than those after RFCA assets by LGE in MRI in 1–3 months after PVI [17]. Moreover, a study by Ruiz-Granell et al. demonstrated that scar formation and the presence of potential conduction gaps within the PV differ significantly between CBA and RFCA, which may influence post-procedural atrial remodeling, supporting the view that different energy sources interact with the atrial substrate in distinct ways [18].

Several studies have investigated the effects of RFCA on LA hemodynamic and mechanical function, evaluating changes both in the acute post-procedural phase and during mid- to long-term follow-up. One study, assessed using cardiac MRI, demonstrates a marked acute deterioration of LA mechanical function following PVI performed with RFCA [19]. In the acute phase, assessed 72 h after PVI using cardiac MRI, a significant deterioration of LA hemodynamic function was observed, reflected by reductions in PALS, contractile strain, and LAEF. At the 3-month follow-up, the authors reported a further reduction in LA size assessed by LAVI, accompanied by an improvement in LA contractile function, with increases in PALS, contractile strain, and LAEF. Importantly, PALS and contractile strain did not return to their baseline values, and their reduction remained statistically significant. The authors suggest that the lack of full strain normalization may be related to irreversible myocardial loss caused by ablation lesions and subsequent fibrotic remodeling. While their findings regarding LA strain are consistent with our observations, we did not observe a reduction in LA dimensions assessed by LAVI during follow-up [19].

Another study invasively analyzed 17 patients to assess the acute effects of direct PVI on LA function. During the procedure, the authors demonstrated that PVs actively contribute to LA filling through contraction of their muscular sleeves, and that antral PVI leads to immediate deterioration of LA reservoir function and LV contractile performance [20]. These findings support the concept of acute atrial stunning induced by RFCA, although it should be noted that invasive measurements in this setting remain scarce. Importantly, evidence from non-invasive imaging modalities indicates that this effect is at least partially reversible over time, with gradual recovery of LA function during follow-up.

Other comparative strain study showed no improvement in LAS-R or LAS-CT strain after RFCA, including at the 3-month follow-up, in contrast to PFA, which is consistent with our observation of persistent lack of PALS recovery after RFCA. Although CBA was not included, the study provides a useful comparison of ablation modalities [21]. Wieczorek et al. focused on the impact of procedural success of RF-PVI in patients with paroxysmal AF, demonstrating that effective ablation is associated with favorable LA remodeling and improvement in selected echocardiographic parameters during follow-up. These results suggest that, in a well-selected population, improvement in LA structure and function can be expected after successful PVI. However, direct comparison with our findings is limited, as our patients had larger LA size assessed by LAVI, were older and had more comorbidities, and included a mixed population of paroxysmal and persistent AF. These differences likely influenced the extent of post-procedural LA remodeling [22].

The impact of CBA on LA hemodynamic and mechanical function has also been evaluated in both the acute phase and during follow-up. Similar conclusions were obtained in CBA studies. In a study of 36 patients undergoing CBA PVI for paroxysmal AF, successful ablation was associated with prevention of progressive LA enlargement and functional decline, whereas patients with AF recurrence exhibited increases in LA size (LAV, LAVI, LA diameter) and deterioration of filling and emptying function [23]. Canpolat et al. reported similar conclusions, demonstrating that also in patients with paroxysmal AF, successful PVI using CBA was associated with reverse LA structural and electrical remodeling, including a reduction in LAVI and improvements in intra- and inter-atrial electromechanical conduction delays at 12 months among those without arrhythmia recurrence. However, CBA did not prevent LA remodeling in patients with recurrent AF [24]. In a direct comparative analysis of atrial remodeling in patients with paroxysmal AF undergoing PVI using CBA or RFCA, no significant differences in LAVI were observed between groups, while a clear reduction in LA dimensions was documented during long-term (3-year) follow-up [25].

Several hypotheses may explain why the remodeling patterns observed after CBA in our cohort differ from those reported in previous studies. Compared with previous studies, our patient population was characterized by larger baseline LA volumetric parameters, including LAVI (44.13 ± 13.19) and LAA (26.13 ± 5.45). Firstly, available literature identifies a LAVI_max_ cut-off value of <34.4 mL/m^2^ as being associated with the most favorable outcomes after AF ablation [26,27]. Secondly, PV ostia and distal PVs are known to enlarge in association with AF and larger LA, such that patients with markedly increased LA volumes tend to have larger PV ostia [28]. Moreover, increased ostial ovality and non-circular PV anatomy, which are more common in enlarged atria, have been shown to be procedurally challenging, associated with less predictable lesion formation and higher AF recurrence rates, potentially contributing to heterogeneous and less durable scar formation [29,30].

Notably, the majority of cited studies focused on patients with paroxysmal AF, whereas in our cohort this subgroup accounted for only 64% of the study population. Patients with persistent AF and larger LA exhibit a higher burden of atrial fibrosis, as evidenced by low-voltage areas identified in approximately 35% of patients with persistent AF compared with about 10% of those with paroxysmal AF, which has been shown to predict poorer ablation outcomes [31,32]. Moreover, paroxysmal AF was more prevalent in the RFCA group; the inclusion of a substantial proportion of patients with non-paroxysmal AF in the CBA group may have contributed to greater atrial remodeling and fibrosis, potentially explaining differences between our results and prior studies focused primarily on paroxysmal AF. In addition, our patients were frequently older (>60 years) and had a higher burden of comorbidities, factors known to be associated with increased LA fibrosis, which may consequently lead to altered atrial remodeling and a modified response to ablation [32]. While the present study focused primarily on echocardiographic remodeling, the clinical implications of different ablation technologies may extend beyond structural cardiac changes. Recent data suggest that ablation energy source may also influence patient-reported outcomes, including quality of life and symptom burden following AF ablation [33].

## 5. Limitations

The principal limitations of this study are the relatively small sample size and the observational, non-randomized design, which limit statistical power and preclude definitive conclusions regarding causal relationships between ablation modality and atrial remodeling. Consequently, the present findings should be considered exploratory and hypothesis-generating. Although repeated-measures ANOVA was performed to better characterize longitudinal remodeling patterns, the limited cohort size restricted the use of extensive multivariable adjustment and predictive modeling due to the risk of overfitting.

In addition, the study evaluated multiple echocardiographic and clinical endpoints across serial follow-up assessments. Given the exploratory nature of the analysis and the absence of formal adjustment for multiple comparisons, isolated statistically significant findings should be interpreted cautiously, as some associations may reflect chance findings rather than robust treatment-related effects.

Importantly, the present analysis was not performed according to an intention-to-treat principle. Patients with AF or atrial flutter recurrence within six weeks preceding follow-up echocardiography were excluded from the final remodeling analysis in order to minimize the direct influence of active arrhythmia on echocardiographic measurements. However, this approach may have introduced attrition bias, as patients with less favorable rhythm outcomes were disproportionately excluded from the final analysis. Consequently, the observed remodeling patterns may not fully reflect the entire ablated population and should be interpreted with caution.

Arrhythmia recurrence was assessed primarily on a clinical basis without continuous rhythm monitoring, which may have resulted in underdetection of asymptomatic AF episodes and inaccurate estimation of AF burden. Furthermore, the study population was heterogeneous with respect to AF phenotype, baseline atrial dimensions, age, and comorbidity burden, all of which are known to influence atrial remodeling. Notably, patients undergoing CBA had a higher prevalence of persistent AF, which may have contributed to the observed differences in remodeling trajectories. Finally, as this was a single-center study, the generalizability of these findings remains limited and requires confirmation in larger prospective multicenter cohorts.

## 6. Conclusions

In our study, patients undergoing CBA demonstrated an increase in LA dimensions compared with those treated with RFCA during the 12-month follow-up, as measured by LA size; however, these changes were not accompanied by deterioration in LA hemodynamic function. Although most previously published studies did not report such a trend, this discrepancy might be explained by differences in patient population, including larger baseline LA size, older age, greater comorbidity burden, and a higher proportion of non-paroxysmal AF in our cohort, which may have influenced post-ablation atrial remodeling. Overall, our results should be considered hypothesis-generating and suggest potentially different remodeling trajectories following CBA and RFCA. Ongoing and future studies involving larger and more homogeneous populations are needed to validate these observations and further clarify the relationship between ablation modality, atrial substrate, and long-term atrial remodeling.

## Figures and Tables

**Table 1:** Baseline clinical characteristics.

	CBA	RFCA	*p*-Value
**Demographics**			
Age (years)	61.03 ± 11.06	60.5 ± 9.48	n.s.
Sex (male, n%)	17/61	10/71	n.s
BMI (kg/m^2^)	28.78 ± 6.67	28.56 ± 4.46	n.s.
**Comorbidities**			
Heart Failure	8/28.6	3/21.4	n.s.
Chronic coronary syndrome	3/10.71	1/7.14	n.s.
Chronic kidney disease	5/17.85	1/7.14	n.s.
Hypertension	19/67.9	10/71.4	n.s.
Diabetes	5/17.85	1/7.14	n.s.
Obesity (BMI > 30 kg/m^2^)	12/42.85	3/21.42	n.s.
**Atrial fibrillation characteristic**			
PAF	17/60.7	12/86	n.s.
CHA_2_DS_2_-VASc score	2.14 ± 1.29	1.71 ± 1.20	n.s.
HAS-BLED score	0.68 ± 0.72	0.71 ± 0.61	n.s.
EHRA score	2.61 ± 0.74	2.86 ± 0.53	n.s.
ECV in history	1.03 ± 1.35	1 ± 2.08	n.s.
AAD in history	0.64 ± 0.73	0.71 ± 0.61	n.s.
History of AF (years)	5.66 ± 4.45	4.93 ± 3.85	n.s.
**Drugs**			
B-blockers	21/75	7/50	n.s
AAD type I	4/14	4/28.6	n.s.
AAD type III	6/21.4	2/14.3	n.s.
DOAC	24/85.7	13/93	n.s.
Apixaban	15/53.6	2/14.3	0.02
Riwaroxaban	9/32	10/71.3	0.02
Dabigatran	3/10.7	1/7.14	n.s.
Vitamin K antagonist	0/0	0/0	n.s.
ACE-I	16/57.15	7/50	n.s.
**Ablation procedure and outcomes**			
Non-responders	4/14.3	6/43	0.06
Day of procedure (day)	1.64 ± 0.49	1.78 ± 0.43	n.s.
Duration of hospitalization (days)	2.89 ± 1.13	2.64 ± 0.63	n.s.

Baseline demographic and clinical characteristics of the study population undergoing pulmonary vein isolation. Continuous variables are described in terms of means and standard deviations, while categorical data are described using percentages and frequencies. Abbreviations: AF—atrial fibrillation; PAF—paroxysmal atrial fibrillation; CBA—cryoablation; RFCA—radiofrequency catheter ablation; BMI—body mass index; AAD—antiarrhythmic drug; DOAC—direct oral anticoagulant; ACE-I—angiotensin-converting enzyme inhibitor; n.s.—not significant.

**Table 2:** Echocardiographic characteristics at baseline between CBA and RFCA group.

	CBA	RFCA	*p*-Value
LA diameter [mm]	43.71 ± 6.55	41.73 ± 4.71	n.s.
LAA [cm^2^]	26.13 ± 5.45	26.14 ± 4.32	n.s.
LAVI (MOD) [mL/m^2^]	42.23 ± 12.30	40.84 ± 11.40	n.s.
LAVI (AL) [mL/m^2^]	44.34 ± 13.19	43.97 ± 11.58	n.s.
LAS-R [%]	19.89 ± 5.40	23.67 ± 8.02	n.s.
LAS-CD [%]	−9.07 ± 3.65	−12.53 ± 5.95	n.s.
LAS–CT [%]	−10.70 ± 3.57	−10.27 ± 3.94	n.s.
LAEF [%]	48.04 ± 10.70	52.73 ± 12.96	n.s.
LAEV [mL]	30.75 ± 10.26	31.13 ± 7.78	n.s.
LAV max [mL]	65.86 ± 20.19	61.27 ± 17.68	n.s.
LAV min [mL]	35.82 ± 15.30	30.2 ± 15.83	n.s.
LAVpreA [mL]	53.71 ± 18.85	46.13 ± 17.27	n.s
LAKE [kndes.cm]	8.37 ± 6.84	6.37 ± 5.43	n.s.
LAFI	20.47 ± 10.54	28.80 ± 12.06	n.s.
LA expansion index [%]	96 ± 48	127 ± 65	n.s.
LA AE [%]	35 ± 12	36 ± 15	n.s.
LA PE [%]	19 ± 12	26 ± 11	0.06
LAS-si [$\frac{cm/s}{\%}$]	0.44 ± 0.24	0.40 ± 0.31	n.s.
E/e’ avr [cm/s]	7.61 ± 3.00	7.77 ± 3.32	n.s.

Echocardiographic parameters of the entire study population assessed before pulmonary vein isolation stratified by CBA and RFCA Abbreviations: CBA—cryoablation; RFCA—radiofrequency catheter ablation; LA—left atrium; LAA—left atrial appendage; LAVI—left atrial volume index; LAVmax—maximal left atrial volume; LAVmin—minimal left atrial volume; LAVpreA—left atrial volume before atrial contraction; LAS-r—left atrial reservoir strain; LAS-cd—left atrial conduit strain; LAS-ct—left atrial contractile strain; LAS-si—left atrial stiffness index; LA EF—left atrial ejection fraction; LA PE—left atrial passive emptying fraction; LA AE—left atrial active emptying fraction; LAFI—left atrial functional index; E/e′—ratio of early mitral inflow velocity to early diastolic mitral annular velocity; n.s.—not significant.

**Table 3:** Baseline and 12-month follow-up echocardiographic characteristics of the CBA and RCA groups.

Parameter	CBA Change (95% CI)	RFCA Change (95% CI)	Group × Time Interaction (*p*)
**LA diameter [mm]**	**+2.50 (1.18 to 3.82)**	**−1.47 (−3.79 to 0.85)**	**0.0017**
LAA [cm^2^]	+2.34 (1.13 to 3.55)	−0.41 (−3.95 to 3.14)	0.0645
LAVI (MOD) [mL/m^2^]	+4.32 (1.58 to 7.07)	+1.40 (−5.00 to 7.80)	0.3089
LAVI (AL) [mL/m^2^]	+5.32 (2.34 to 8.31)	+1.06 (−6.33 to 8.45)	0.1901
LAS-R [%]	−0.18 (−2.16 to 1.81)	−3.93 (−7.95 to 0.08)	0.0547
LAS-CD [%]	−0.21 (−2.32 to 1.89)	+2.60 (−0.47 to 5.67)	0.1155
LAS–CT [%]	+1.57 (−0.36 to 3.51)	+0.13 (−3.13 to 3.40)	0.4027
LAEF [%]	−3.04 (−7.86 to 1.79)	−0.97 (−6.34 to 4.40)	0.5798
LAEV [mL]	+1.57 (−1.99 to 5.13)	+0.40 (−4.17 to 4.97)	0.6819
LAV max [mL]	+6.07 (0.54 to 11.60)	+0.20 (−7.92 to 8.32)	0.2097
LAV min [mL]	+5.43 (0.47 to 10.39)	−0.67 (−5.90 to 4.56)	0.1130
LAVpreA [mL]	+4.39 (−0.72 to 9.50)	+0.07 (−5.40 to 5.53)	0.2722
LA expansion index [%]	−9 (−27 to 9)	−9 (−35 to 18)	0.9992
LA-AE [%]	−5 (−12 to 2)	+1 (−7 to 9)	0.3219
LA-PE [%]	+0 (−3 to 4)	−2 (−7 to 4)	0.4802
LAFI	−5.06 (−9.65 to −0.46)	−3.89 (−10.25 to 2.46)	0.7583
LAKE [kndes.cm]	+7310 (−18,760 to 33,380)	−14,842 (−33,888 to 4205)	0.2406

Data are presented as mean changes from baseline with 95% confidence intervals. *p*-values represent the group-by-time interaction obtained from repeated-measures ANOVA. Statistically significant findings are shown in bold. Abbreviations: LA—left atrium; LAA—left atrial appendage; LAVI—left atrial volume index; LAVmax—maximal left atrial volume; LAVmin—minimal left atrial volume; LAVpreA—left atrial volume before atrial contraction; LAS-r—left atrial reservoir strain; LAS-cd—left atrial conduit strain; LAS-ct—left atrial contractile strain; LA EF—left atrial ejection fraction; LA PE—left atrial passive emptying fraction; LA AE—left atrial active emptying fraction; LAFI—left atrial functional index; n.s.—not significant.

## Data Availability

The data supporting the report are available from Jonasz Kozielski (jonaszkozielski@gmail.com) on reasonable request.

## Abbreviations

The following abbreviations are used in this manuscript:

AF	Atrial fibrillation
LA	Left atrium
CBA	Cryoablation
RFCA	Radiofrequency catheter ablation

## References

[1] Stewart S., Hart C.L., Hole D.J., McMurray J.J. (2001). Population prevalence, incidence, and predictors of atrial fibrillation in the Renfrew/Paisley study. Heart.

[2] Hindricks G., Potpara T., Dagres N., Arbelo E., Bax J.J., Blomström-Lundqvist C., Boriani G., Castella M., Dan G.A., Dilaveris P.E. (2021). 2020 ESC Guidelines for the diagnosis and management of atrial fibrillation developed in collaboration with the European Association for Cardio-Thoracic Surgery (EACTS): The Task Force for the diagnosis and management of atrial fibrillation of the European Society of Cardiology (ESC) Developed with the special contribution of the European Heart Rhythm Association (EHRA) of the ESC. Eur. Heart J..

[3] Goette A., Corradi D., Dobrev D., Aguinaga L., Cabrera J.A., Chugh S.S., de Groot J.R., Soulat-Dufour L., Fenelon G., Hatem S.N. (2024). Atrial cardiomyopathy revisited-evolution of a concept: A clinical consensus statement of the European Heart Rhythm Association (EHRA) of the ESC, the Heart Rhythm Society (HRS), the Asian Pacific Heart Rhythm Society (APHRS), and the Latin American Heart Rhythm Society (LAHRS). Europace.

[4] Tzeis S., Gerstenfeld E.P., Kalman J., Saad E.B., Sepehri Shamloo A., Andrade J.G., Barbhaiya C.R., Baykaner T., Boveda S., Calkins H. (2024). 2024 European Heart Rhythm Association/Heart Rhythm Society/Asia Pacific Heart Rhythm Society/Latin American Heart Rhythm Society expert consensus statement on catheter and surgical ablation of atrial fibrillation. Europace.

[5] Masuda M., Sekiya K., Asai M., Iida O., Okamoto S., Ishihara T., Nanto K., Kanda T., Tsujimura T., Matsuda Y. (2022). Influence of catheter ablation for atrial fibrillation on atrial and ventricular functional mitral regurgitation. ESC Heart Fail..

[6] Yoshida K., Yui Y., Kimata A., Koda N., Kato J., Baba M., Misaki M., Abe D., Tokunaga C., Akishima S. (2014). Troponin elevation after radiofrequency catheter ablation of atrial fibrillation: Relevance to AF substrate, procedural outcomes, and reverse structural remodeling. Heart Rhythm.

[7] Packer M. (2019). Effect of catheter ablation on pre-existing abnormalities of left atrial systolic, diastolic, and neurohormonal functions in patients with chronic heart failure and atrial fibrillation. Eur. Heart J..

[8] Kuck K.H., Fürnkranz A., Chun K.J., Metzner A., Ouyang F., Schlüter M., Elvan A., Lim H.W., Kueffer F.J., Arentz T. (2016). Cryoballoon or radiofrequency ablation for symptomatic paroxysmal atrial fibrillation: Reintervention, rehospitalization, and quality-of-life outcomes in the FIRE AND ICE trial. Eur. Heart J..

[9] Schmidt M., Marschang H., Clifford S., Harald R., Guido R., Oliver T., Johannes B., Daccarett M. (2012). Trends in inflammatory biomarkers during atrial fibrillation ablation across different catheter ablation strategies. Int. J. Cardiol..

[10] Hernández-Romero D., Marín F., Roldán V., Peñafiel P., Vilchez J.A., Orenes-Piñero E., Giner J.A., Valdés M., García-Alberola A. (2013). Comparative determination and monitoring of biomarkers of necrosis and myocardial remodeling between radiofrequency ablation and cryoablation. Pacing Clin. Electrophysiol..

[11] Malmborg H., Christersson C., Lönnerholm S., Blomström-Lundqvist C. (2013). Comparison of effects on coagulation and inflammatory markers using a duty-cycled bipolar and unipolar radiofrequency pulmonary vein ablation catheter vs. a cryoballoon catheter for pulmonary vein isolation. Europace.

[12] Antolič B., Pernat A., Cvijić M., Žižek D., Jan M., Šinkovec M. (2016). Radiofrequency catheter ablation versus balloon cryoablation of atrial fibrillation: Markers of myocardial damage, inflammation, and thrombogenesis. Wien. Klin. Wochenschr..

[13] Kühne M., Suter Y., Altmann D., Ammann P., Schaer B., Osswald S., Sticherling C. (2010). Cryoballoon versus radiofrequency catheter ablation of paroxysmal atrial fibrillation: Biomarkers of myocardial injury, recurrence rates, and pulmonary vein reconnection patterns. Heart Rhythm.

[14] Herrera Siklódy C., Arentz T., Minners J., Jesel L., Stratz C., Valina C.M., Weber R., Kalusche D., Toti F., Morel O. (2012). Cellular damage, platelet activation, and inflammatory response after pulmonary vein isolation: A randomized study comparing radiofrequency ablation with cryoablation. Heart Rhythm.

[15] Khan I.A. (2002). Transient atrial mechanical dysfunction (stunning) after cardioversion of atrial fibrillation and flutter. Am. Heart J..

[16] Khan I.A. (2003). Atrial stunning: Basics and clinical considerations. Int. J. Cardiol..

[17] Kurose J., Kiuchi K., Fukuzawa K., Mori S., Ichibori H., Konishi H., Taniguchi Y., Hyogo K., Imada H., Suehiro H. (2018). The lesion characteristics assessed by LGE-MRI after the cryoballoon ablation and conventional radiofrequency ablation. J. Arrhythmia.

[18] Ruiz-Granell R., Ballesteros G., Andreu D., Erkiaga A., Ferrero-De-Loma-Osorio A., Ramos P., Martínez-Brotons A., Vives-Rodríguez E., Izquierdo-de-Francisco M., García-Bolao I. (2019). Differences in scar lesion formation between radiofrequency and cryoballoon in atrial fibrillation ablation: A comparison study using ultra-high-density mapping. Europace.

[19] Pouderoijen N.V., Hopman L.H.G.A., Wentrup L.E., de Groot J.R., Kemme M.J.B., Allaart C.P., Götte M.J.W. (2025). Left atrial volumetric and functional remodeling post-pulmonary vein isolation: Insights from cardiac magnetic resonance imaging. J. Cardiovasc. Magn. Reson..

[20] Mamchur S., Mamchur I., Khomenko E., Kokov A., Bokhan N., Sherbinina D. (2014). Mechanical function of left atrium and pulmonary vein sleeves before and after their antrum isolation. Medicina.

[21] Verhaeghe L., L’Hoyes W., Vijgen J., Phlips T., Hoffmann R., Ferreira S.M., Stassen J., Herbots L., Dendale P., Falter M. (2025). Left atrial function after atrial fibrillation ablation with pulsed field vs. radiofrequency energy: A comparative study. Heart Rhythm.

[22] Wieczorek J., Mizia-Stec K., Cichoń M., Wieczorek P., Woźniak-Skowerska I., Hoffmann A., Wnuk-Wojnar A.M., Szydło K. (2023). Positive left atrial remodeling in patients with paroxysmal atrial fibrillation after a successful radiofrequency pulmonary vein isolation. Kardiol. Pol..

[23] Erdei T., Dénes M., Kardos A., Mihálcz A., Földesi C., Temesvári A., Lengyel M. (2012). Could successful cryoballoon ablation of paroxysmal atrial fibrillation prevent progressive left atrial remodeling?. Cardiovasc. Ultrasound.

[24] Canpolat U., Aytemir K., Özer N., Oto A. (2015). The impact of cryoballoon-based catheter ablation on left atrial structural and potential electrical remodeling in patients with paroxysmal atrial fibrillation. J. Interv. Card. Electrophysiol..

[25] Wang X., Song B., Qiu C., Han Z., Wang X., Lu W., Chen X., Chen Y., Pan L., Sun G. (2021). The effect of left atrial remodeling after cryoballoon ablation and radiofrequency ablation for paroxysmal atrial fibrillation. Clin. Cardiol..

[26] Albano A.J., Bush J., Parker J.L., Corner K., Lim H.W., Brunner M.P., Dahu M.I., Dandamudi S., Elmouchi D., Gauri A. (2019). Left Atrial Volume Index Predicts Arrhythmia-Free Survival in Patients with Persistent Atrial Fibrillation Undergoing Cryoballoon Ablation. J. Atr. Fibrillation.

[27] Zhuang J., Wang Y., Tang K., Li X., Peng W., Liang C., Xu Y. (2012). Association between left atrial size and atrial fibrillation recurrence after single circumferential pulmonary vein isolation: A systematic review and meta-analysis of observational studies. Europace.

[28] Schwartzman D., Lacomis J., Wigginton W.G. (2003). Characterization of left atrium and distal pulmonary vein morphology using multidimensional computed tomography. J. Am. Coll. Cardiol..

[29] Schmidt M., Dorwarth U., Straube F., Daccarett M., Rieber J., Wankerl M., Krieg J., Leber A.W., Ebersberger U., Huber A. (2013). Cryoballoon in AF ablation: Impact of PV ovality on AF recurrence. Int. J. Cardiol..

[30] Stachyra M., Szczasny M., Tarkowski A., Mianowana M., Wojewoda K., Wysokinska K., Blaszczak P., Głowniak A. (2021). Impact of pulmonary vein ovality index on cooling kinetics and acute success of atrial fibrillation ablation with the third-generation cryoballoon catheter. Postępy Kardiol. Interwencyjnej.

[31] Marrouche N.F., Wilber D., Hindricks G., Jais P., Akoum N., Marchlinski F., Kholmovski E., Burgon N., Hu N., Mont L. (2014). Association of atrial tissue fibrosis identified by delayed enhancement MRI and atrial fibrillation catheter ablation: The DECAAF study. J. Am. Med. Assoc..

[32] Rolf S., Kircher S., Arya A., Eitel C., Sommer P., Richter S., Gaspar T., Bollmann A., Altmann D., Piedra C. (2014). Tailored atrial substrate modification based on low-voltage areas in catheter ablation of atrial fibrillation. Circ. Arrhythmia Electrophysiol..

[33] Matteucci A., Russo M., Galeazzi M., Pandozi C., Bonanni M., Mariani M.V., Pierucci N., La Fazia V.M., Di Fusco S.A., Nardi F. (2025). Impact of Ablation Energy Sources on Perceived Quality of Life and Symptom in Atrial Fibrillation Patients: A Comparative Study. J. Clin. Med..

